# Early pullout of lateral row knotless anchor in rotator cuff repair

**DOI:** 10.4103/0973-6042.59972

**Published:** 2009

**Authors:** Chrysi Tsiouri, Daniel H Mok

**Affiliations:** Epsom Hospital, Epsom, Surrey, United Kingdom

**Keywords:** Knotless anchors, lateral anchor, osteoporosis

## Abstract

Use of lateral row anchors in rotator cuff repair as a means of enhancing the strength of the repair; and improving footprint tendon contact, thus promoting healing, is becoming more popular in current arthroscopic practice. In our knowledge, failures of lateral row knotless anchors have not yet been reported. We present a case of double row rotator cuff repair using a Swivelock anchor (Arthrex) as a lateral row anchor that failed two weeks after surgery.

## INTRODUCTION

Various surgical techniques are described in literature that attempt to recreate the anatomical rotator cuff footprint when performing an arthroscopic rotator cuff repair, with double row repairs being the subject of controversy. We report the failure of a lateral knotless anchor.

## CASE REPORT

A fifty nine year old female was admitted for repair of her non dominant left rotator cuff tear. Arthroscopy revealed a Superior Labrum Anterior Posterior (SLAP) lesion, which was unstable as well as a full-thickness tear of the supraspinatus tendon, completely off the attachment at the greater tuberosity.

**Figure 1:**
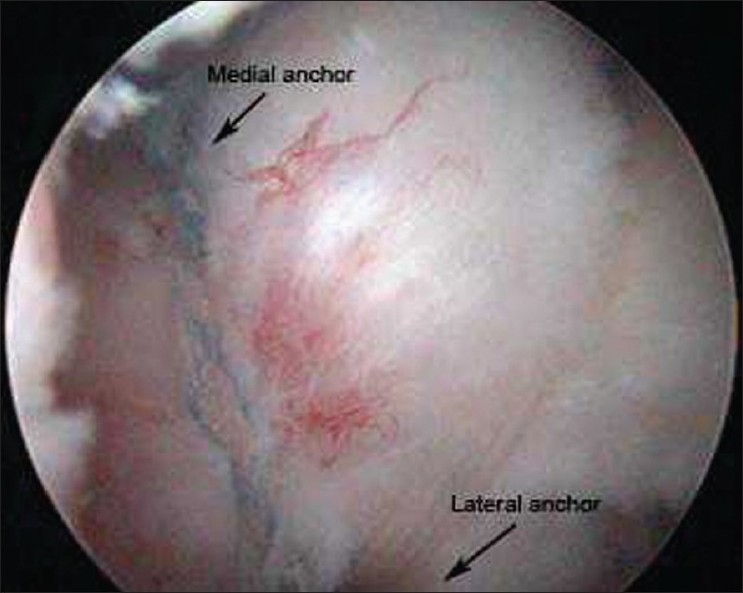
Double row rotator cuff repair with good contact of tendon onto footprint

The SLAP tear was secured with simple sutures via a 3.5 mm absorbable copolymer suture anchor (Lactoscrew, BIOMET) inserted at the 12-o'clock position behind the long head of biceps. The torn supraspinatus tendon was first secured with two sets of mattress sutures from the medial 5.5 mm absorbable copolymer suture anchor (Lactoscrew, BIOMET) that was inserted in the articular margin of the greater tuberosity. All four strands of the two sutures of the medial anchor were then thread through a second row 5.5 mm Poly-(L-lactide) (PLLA) knotless anchor (Swivelock, Arthrex) that was inserted into the lateral wall of the tuberosity cortex, 1 cm away from the first anchor [Figure 1]. The shoulder was immobilized in a sling for 2 weeks, after which physiotherapy was started.

At that time, she developed a painful red swelling in her axilla resembling an inflamed lymph node, but there were no clinical or biochemical signs of infection. Shoulder movements, though painful, were not restricted. A crepitus, however, was felt in the subacromial space on rotation. Ultrasound examination revealed one of the anchors had backed out. An urgent MRI scan confirmed that the lateral row anchor had migrated.

**Figure 2:**
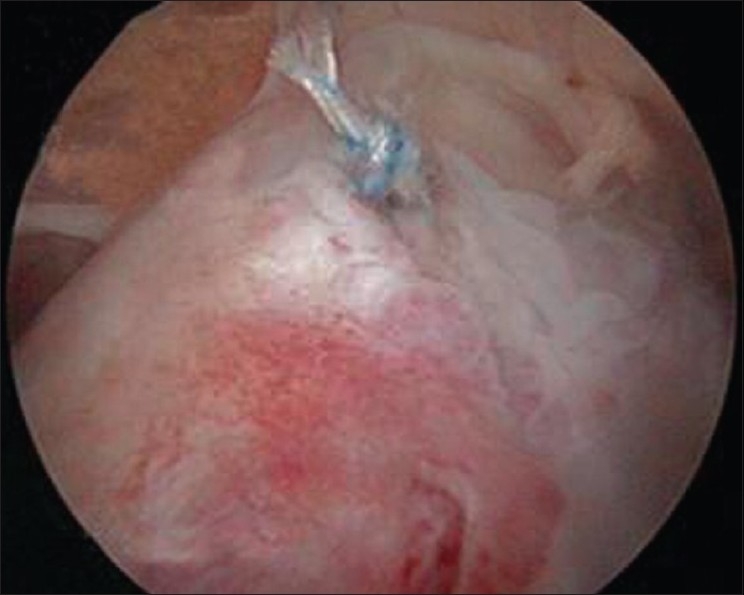
Medial anchor is strong enough to maintain the repair integrity on its own

**Figure 3:**
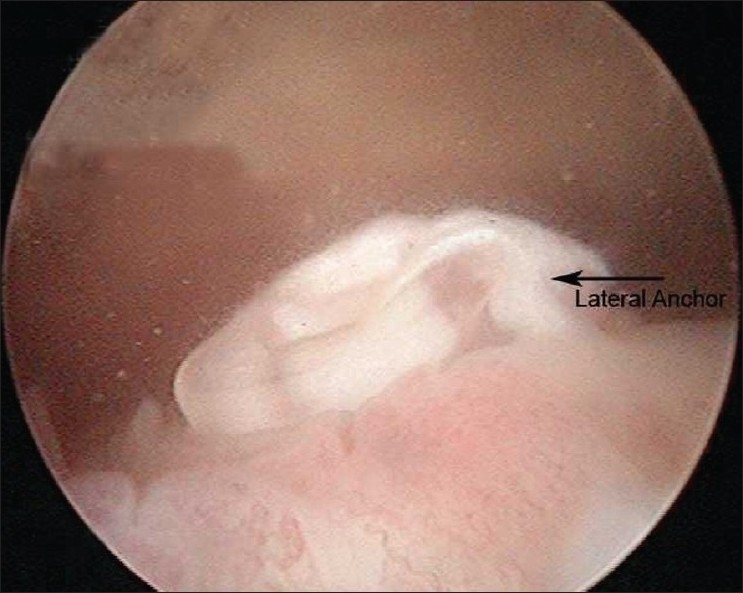
The PLLA part of the lateral anchor has been pulled out and is protruding

**Figure 4:**
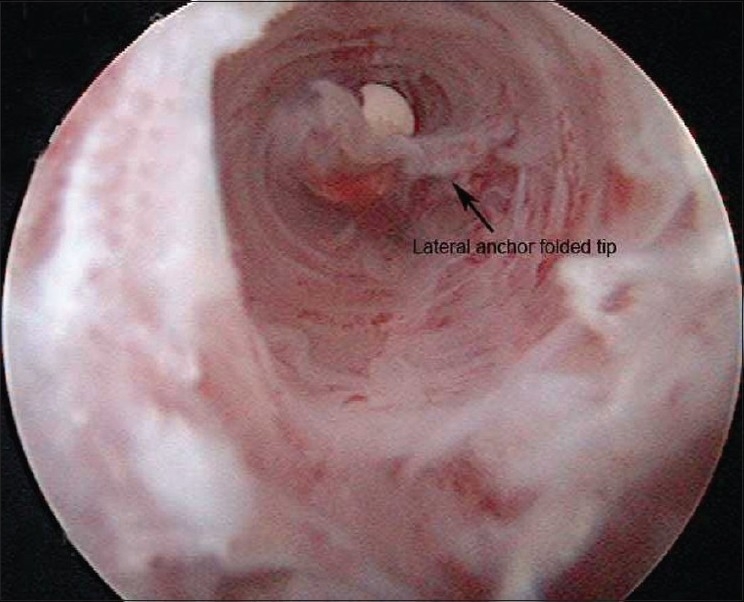
The peek-forked tip of the lateral anchor is in the threaded anchor hole. Threads filled with fibrous tissue can be seen

Subsequent repeat arthroscopy showed that a good repair of the supraspinatus tendon was achieved with the medial anchor [Figure 2]. The lateral row anchor was prominent above the lateral cortex [Figure 3]. The protruding anchor and the redundant sutures were removed [Figure 4].

## DISCUSSION

As arthroscopic rotator cuff repair is more commonly practiced,[1,2] the importance of restoring the anatomical footprint to enhance healing is emphasized.[1,3] Double row tendon repair is an accepted method of recreating the cuff footprint,[1,3,4] biomechanically stronger,[5] and it offers higher structural integrity.[5] There is, however, no evidence that these advantages have clinical implications.[6] Meanwhile, the introduction of knotless anchors as lateral row fixation or suture bridge devices facilitates the double row technique. Biomechanical testings have shown that these 'push in' anchors have equivalent pullout strength when compared with the traditional suture anchors in this situation. The Swivelock was the latest knotless design with the highest pullout strength at 712 N. Its failure mode is secondary only to eyelet or suture breakage but not due to anchor pulled out.[7]

We hypothesized that the anchor failed in our case for the following reasons.

After a mattress repair to the torn tendon, we placed all four strands of sutures from the single medial anchor to be carried by the lateral row anchor. When the Swivelock was screwed into position, it may have generated high tension in the sutures between the two anchors. When the tendon started to contract with mobilization, all the traction forces on the medial anchor were then transmitted across to the lateral anchor. This tension may be beyond the level of tension that the bone of the lateral cortex can withstand. Whereas if it was just one set of sutures, only part of the tension would be transmitted to the lateral anchor. Further biomechanical testing of this hypothesis is needed.

Secondly, the effectiveness of the device is directly related to the quality of bone into which it is inserted. Reduced bone density of the tuberosity secondary to disuse, e.g., after injury, may have contributed to a weak fixation.[8] In addition, it is well recognized that the pullout strength of anchors is reduced in osteoporotic bone.[8] With the mean age of the patients requiring arthroscopic repair of their rotator cuffs rising, we are likely to come across patients with some degree of osteopenia in their greater tuberosity more often.

We therefore recommend that caution should be exercised when placing lateral anchors into greater tuberosities with suspected osteopenia, particularly in cases of postmenopausal female patients whose medial anchor insertion does not inspire secure fixation. In addition, we should not put all four strands of suture from the same medial anchor into the lateral anchor in double row fixation.
